# Vision and Foraging in Structurally Complex Habitats: Common Moorhens (*Gallinula chloropus*) See the Way Ahead

**DOI:** 10.1002/ece3.74060

**Published:** 2026-08-27

**Authors:** Steven J. Portugal, Eleanor A. Lucas, Simon Potier, Gérard Rocamora, Graham R. Martin

**Affiliations:** ^1^ Department of Biological Sciences The University of Oxford Oxford UK; ^2^ Department of Biological Sciences, School of Life and Environmental Sciences Royal Holloway, University of London Egham Surrey UK; ^3^ Les Ailes de l’Urga Marcilly la Campagne France; ^4^ Simon Potier Expert Scientifique Marcilly la Campagne Foucrainville France; ^5^ Island Conservation Society Mahé Seychelles; ^6^ Island Biodiversity and Conservation Centre Université of Seychelles Mahé Seychelles; ^7^ School of Biosciences The University of Birmingham Edgbaston UK

**Keywords:** avian, binocularity, Gruiformes, Rallidae, sensory ecology, visual fields

## Abstract

This study quantifies the visual field configuration of common moorhens ( *Gallinula chloropus* ) to examine how sensory systems balance the competing demands of foraging and predator detection in structurally complex habitats. Using the ophthalmoscopic reflex method, we show that moorhens possess a vertically elongated but relatively narrow binocular field (maximum width ~34°, vertical extent 125°), coupled with extensive monocular coverage producing a near‐panoramic cyclopean field (~300°). This configuration aligns with patterns observed in visually guided foragers and is consistent with a role in precise bill control during close‐range feeding, while maintaining wide lateral and posterior coverage for predator detection. Visual access to the substrate is posture‐dependent, requiring head pitching during foraging, indicating dynamic modulation of sensory input. Comparisons with blue cranes (*Grus paradisea*) highlight ecological, rather than phylogenetic, drivers of visual field variation. These findings provide novel baseline data for Rallidae and reinforce the role of sensory trade‐offs in shaping avian ecological performance.

## Graphical abstract

This study examines how common moorhens balance foraging and predator detection through their visual field configuration. It finds they have a narrow but vertically elongated binocular field for precise feeding, alongside near‐panoramic monocular vision for wide environmental awareness. Their ability to see the ground depends on head movement, showing that visual input is actively adjusted during foraging. Comparisons with other species suggest that ecological needs, rather than evolutionary lineage, primarily shape these visual adaptations.

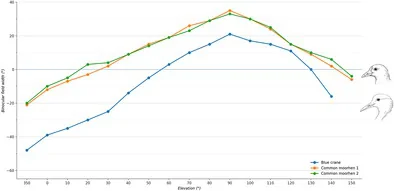

## 1 Introduction

Vision provides animals with rapid and spatially precise information about their surroundings and plays a central role in shaping behaviour, ecological interactions and survival. In birds, vision underpins key functions including navigation, predator detection, social interactions and the control of foraging movements (Martin 2007, 2017a). However, the effectiveness of visual information depends critically on whether stimuli fall within the bird's visual field at any given moment. Avian visual fields describe the three‐dimensional space around the head from which visual information can be extracted instantaneously and are conventionally divided into monocular regions, a binocular region where the visual fields of both eyes overlap, and blind areas where no vision is available (Martin 2007). Together, these components define the cyclopean field (the total visual field produced by the combination of both monocular fields) and represent a fundamental element of avian sensory ecology (Fernández‐Juricic et al. 2008; Martin 2007). Substantial interspecific variation exists in the size, shape and orientation of these regions, resulting from anatomical differences in eye placement, cranial morphology and eye movements (Martin 2017a; Portugal et al. 2023). Importantly, visual field configuration constrains what visual information can be available to a bird at any moment, thereby linking sensory design directly to function through the behavioural and perceptual challenges that a species faces in its natural environment (Martin 2014, 2017b). Visual field configuration in birds is influenced by ecological factors such as habitat complexity, predation pressure and foraging behaviour. Species occupying densely vegetated habitats or relying on visually guided foraging may face different visual demands, resulting in variation in binocular field shape and overall visual coverage.

Two primary functions of avian vision have been widely recognised: the detection of predators and conspecifics at distance, and the accurate control of the bill or feet during close‐range tasks such as foraging, chick provisioning and nest construction (Fernández‐Juricic et al. 2008; Lamond et al. 2025; Martin 2014). These functions place contrasting informational demands on the visual system. Predator detection relies largely on information from lateral and posterior fields, favouring wide monocular coverage and large cyclopean fields, whereas precise control of the bill or talons depends on information from the binocular field (Martin 2014, 2017b; Portugal et al. 2017). The vertical elongation of the binocular field provides visual information across a broad range of elevations in front of the head, facilitating accurate control of head and bill movements by allowing the bird to compare visual motion on either side of the bill when directing and timing bill contact. A familiar behavioural example of this function is adult chick provisioning in moorhens, where adults transfer individual food items directly from their own bill to the bill of a begging chick. Successful food transfer requires precise alignment and timing of bill movements, illustrating the importance of binocular vision for close‐range bill control. Similarly, accurate binocular guidance is required during pecking at food items while foraging. The binocular field provides symmetrical optic flow cues that allow birds to estimate both the direction of movement towards a target and the timing of contact (Martin 2009, 2017b). As a result, visual field design is shaped by a trade‐off between panoramic coverage and binocular overlap (Martin 2017b). Comparative studies across a wide range of avian taxa have shown that this trade‐off is resolved primarily in relation to foraging ecology rather than phylogenetic history or the demands of locomotion (Martin 1999, 2017b; Potier et al. 2018, 2023; Cantlay et al. 2019, 2023).

Despite the strong evidence linking visual field configuration to foraging ecology, relatively few studies have examined visual fields in species that combine visually guided foraging with frequent use of structurally complex habitats. Rails, crakes, and coots (Rallidae, Gruiformes) (Gill et al. 2025) represent informative taxa in this respect. They are semi‐aquatic birds that exploit a wide range of freshwater habitats, including reedbeds, vegetated margins and open water, where they forage on a diverse diet of plant material and invertebrates and are capable of long‐distance dispersal (Cramp and Simmons 1980). Many species live much of their lives in dense and cluttered habitats. Foraging is typically conducted at close range using pecking and grasping actions, often while moving through dense vegetation. These conditions are likely to impose competing simultaneous visual demands; for predator detection and precise bill control for prey capture and chick feeding (Martin et al. 2005).

While the diets of Rallidae are omnivorous, most of their diet does not consist of agile or evasive items but nevertheless requires accurate control of bill position and timing using cues from the binocular field (Cramp and Simmons 1980; Garnett 1978). Even when foraging in cluttered habitats, foraging Rallidae are exposed to predation risk from both aerial and terrestrial predators including birds, mammals, reptiles and fish (Alvarez 1993; Alvarez et al. 2006), and this may favour extensive lateral and posterior visual coverage (Martin 2017b). How these potentially conflicting demands are resolved in the visual field configuration of Rallidae is unknown. In this study, we examine the visual field configuration of common moorhens ( *Gallinula chloropus* ) to determine whether they exhibit the general characteristics associated with visually guided foraging (*sensu* Martin 2014), and to assess how binocular, monocular, and blind regions are arranged. By situating moorhen visual fields within the broader comparative framework of avian sensory ecology, this study contributes to a more complete understanding of how visual systems are adapted to balance the competing demands of foraging and predator detection.

## 2 Methods

Fieldwork was conducted at Aride Island Nature Reserve, Seychelles (4.214° S, 55.668° E) in June 2023. Two adults of the *orientalis* subspecies were measured. Visual fields are typically measured in 2–5 individuals (Martin 2017a). It has been shown in domestic homing pigeons ( *Columba livia* ) that visual fields are generally repeatable at the individual and species level (Lucas and Portugal 2025), although the study highlights that caution should be applied when looking at certain elevations, and higher sample sizes should be used when and where available. Visual field measurements were carried out in a darkened room in the early morning to avoid the high temperatures that typically develop in the afternoon in this location. Each bird was released at or close to its capture location within 65 min of capture. Transport from the capture site to the measurement facility did not exceed 10 min. Moorhens were caught by providing crumbled biscuit and bread under a tilted box attached to a piece of string, with the string being pulled once a bird was under the tilted box.

Visual field configuration was quantified using the ophthalmoscopic reflex method, following established protocols described in earlier work (Martin et al. 2007; Martin and Wanless 2015; Cantlay et al. 2020). During measurements, each bird was restrained with the body supported in a foam cradle, while the bill was secured in a species‐specific holder so that the head adopted its natural resting posture. This set‐up ensured a consistent alignment of the head relative to the coordinate system used for visual field mapping (Martin et al. 2007). The technique has been applied across a broad taxonomic range of birds and allows robust comparisons of visual field topography among species (Martin 2017a, 2017b).

**FIGURE 1:**
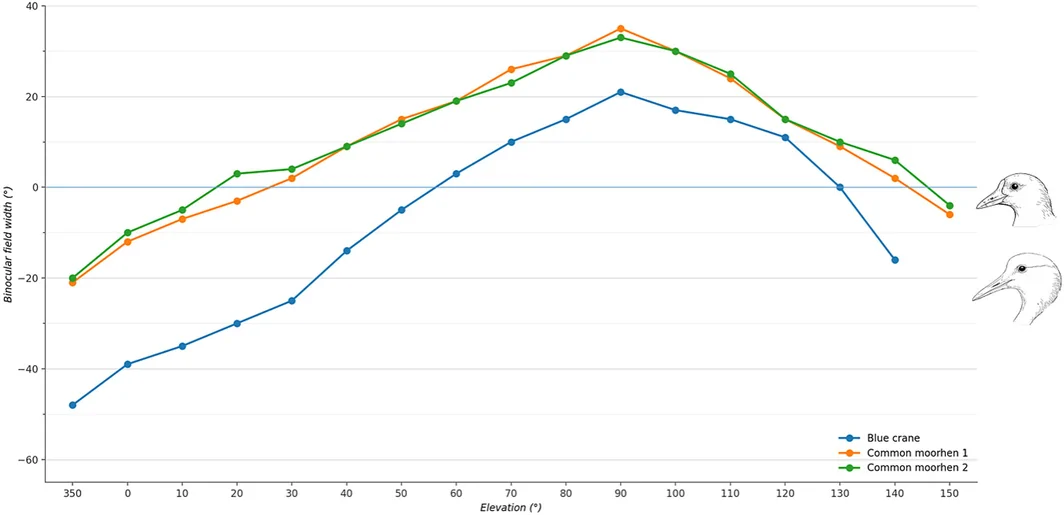
Mean angular separation between the margins of the retinal fields in the anterior visual field is shown as a function of elevation in the head's median sagittal plane. Positive values represent binocular overlap, negative values denote the width of the blind area. In this coordinate system, the horizontal plane corresponds to elevations +90° (anterior to the head), with 0° positioned directly above the head. Data on blue cranes taken from Martin and Shaw (2010). Images of blue crane and common moorhens traced from author photographs.

**FIGURE 2:**
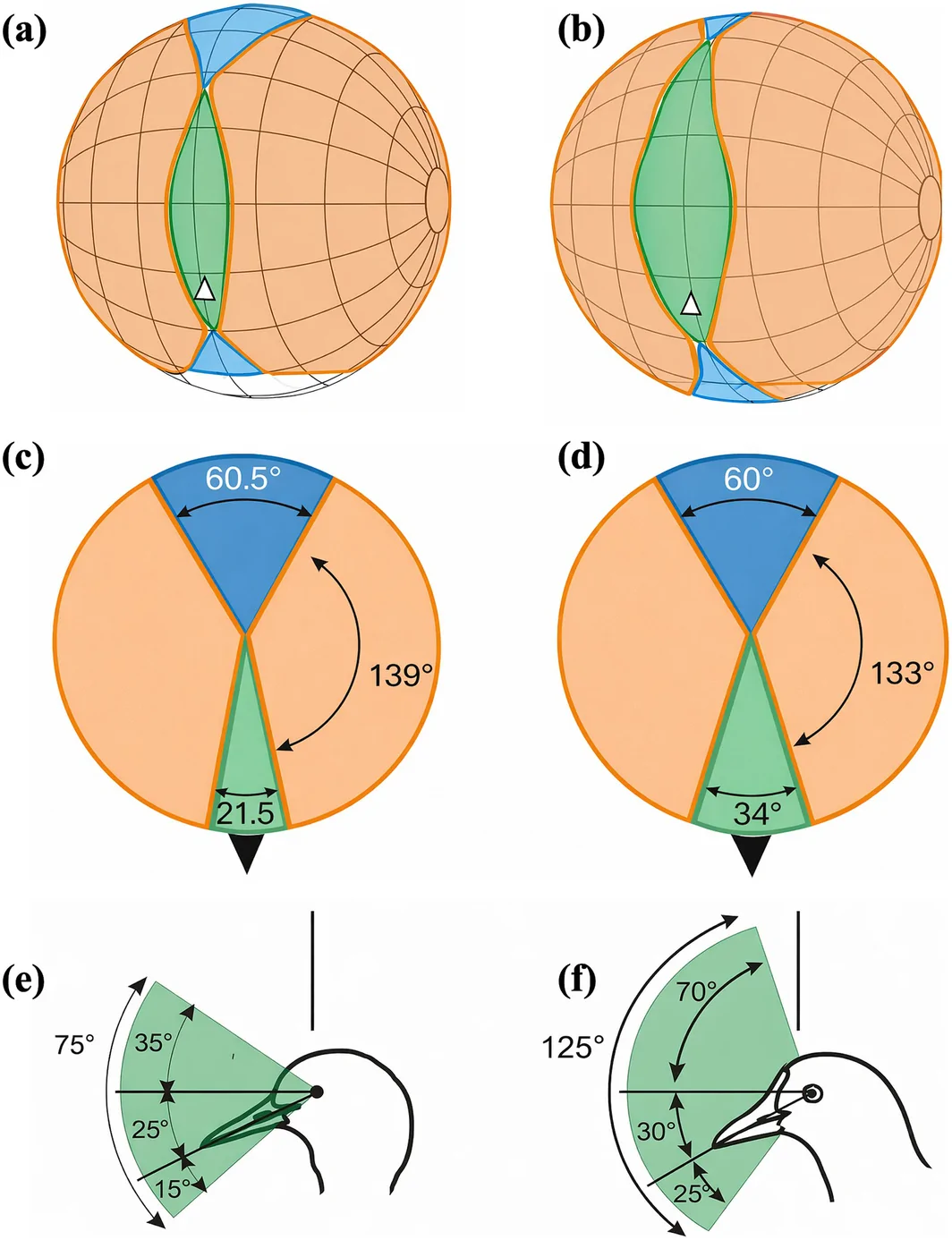
Visual fields of blue cranes (left panel) and common moorhens (right panel). (a, b) Orthographic projections of the visual fields. Visual field maps are presented using a standard latitude–longitude coordinate system, with the equator aligned vertically in the head's median sagittal plane. Gridlines are at 20° intervals. The head is conceptualised as positioned at the centre of a sphere, with visual field boundaries projected onto the surface of the sphere. (c, d) Horizontal sections through the visual fields, the position of the horizontal plane is indicated by the horizontal line in the visual field maps. (e, f) Vertical sections of the binocular fields in the median sagittal plane of the head. Colours denote monocular (orange), binocular (green) and blind (blue) regions of the visual field. The white triangle in (a) (b) and black triangle in (c, d) represents the bill and indicates the direction of the eye and bill‐tip projections.

The procedures were non‐invasive and involved brief restraint, typically not exceeding 30 min; consequently, they did not fall within the scope of the UK Animals (Scientific Procedures) Act 1986 (Martin and Portugal 2011). To comprehensively describe the binocular field, sampling focused on the anterior region of the visual field. An additional measurement directly posterior to the head in the horizontal was made to determine the extent of the cyclopean field in the horizontal plane and from this the width of the blind area and the extent of monocular coverage on either side of the head in the horizontal plane were determined. In total, 17 sagittal measurements plus 1 posterior horizontal measurement were taken per bird. Mean values were calculated (Figure 1) and used to generate a map of the anterior sector of the visual field, and vertical and horizontal cross‐sections through the field (Figure 2).

The results presented are descriptive. For comparative purposes, data from blue cranes (*Grus paradisea*; Gruidae) are included (Martin and Shaw 2010). Blue cranes are the only other species from within the Gruiformes for which visual field data are available.

## 3 Results

In common moorhens, the binocular field is elongated vertically but narrow in width reaching a maximum binocular width of approximately 34° and spanning 125° in vertical extent (Figures 1 and 2; Table 1). The projection of the eye bill‐tip is 30° below the maximum binocular field width, and the maximum width occurs at the horizontal (Figure 1). Monocular field widths average 133° on both sides of the head in the horizontal plane, and the visual field width of each eye is 167° (Figures 1 and 2). The cyclopean visual field extends 300° horizontally, meaning that there is a 60° blind area directly behind the head in the horizontal plane (Figure 2).

**TABLE 1:** Visual field measurements for two common moorhens.

Elevation (°)	Bird 1			Bird 2		
	Right eye	Left eye	Binocularity (°)	Right eye	Left eye	Binocularity (°)
−10	80	101	−21	83	102	−20
0	85	97	−12	87	97	−10
10	88	95	−7	88	93	−5
20	90	93	−3	90	87	3
30	92	90	2	91	87	4
40	96	87	9	97	88	9
50	99	84	15	97	83	14
60	101	82	19	99	80	19
70	105	79	26	101	78	23
80	106	77	29	104	75	29
90	110	75	35	107	74	33
100	107	77	30	104	74	30
110	103	79	24	102	77	25
120	99	84	15	97	82	15
130	95	86	9	95	85	10
140	91	89	2	93	87	6
150	90	96	−6	89	93	−4

## 4 Discussion

The visual field configuration of common moorhens conforms closely to the general pattern described for visually guided foragers (Cantlay et al. 2023).

### 4.1 Configuration of the Binocular Field

The binocular field is vertically elongated yet relatively narrow in horizontal width, with maximum overlap occurring near the horizontal and the projection of the bill positioned below the midpoint of the binocular region (Figure 2). This arrangement is consistent with species that rely on visual information to control precise bill movements during pecking and grasping at non‐evasive prey (Cantlay et al. 2024; Lucas et al. 2024). Wider binocular fields are typically associated with taking evasive prey, such as those seen in raptors (Martin et al. 2012; Potier et al. 2018, 2023; Portugal et al. 2023) pursuit foraging diving ducks (Cantlay et al. 2023), and gulls and terns (Cantlay et al. 2024; Lucas et al. 2024). The vertical elongation of the binocular field provides visual information across a broad range of elevations in front of the head, facilitating accurate control of head and bill movements by allowing the bird to compare visual motion on either side of the bill when directing and timing bill contact. In moorhens, this configuration is consistent with the visual requirements of fine‐scale bill control that is required in this species during chick provisioning (Garnett 1978). Similar precise contact between adult and chick bills in food transfer has been shown to shape the configuration of the binocular field in filter‐feeding flamingos (Martin et al. 2005). Young moorhens do not typically feed independently until they are at least 10 days old. Prior to this stage, adults provision the chicks directly by transferring individual food items from their own bills to those of the young (Garnett 1978). For adults, the fine‐scale bill control is needed in close‐range foraging on the wide range of food items that make up their diet (Cramp and Simmons 1980).

### 4.2 Configuration of the Cyclopean Field

At the same time as facilitating precise bill control, the visual field of moorhens provides extensive lateral and posterior visual coverage. The cyclopean field extends to 300° in the horizontal plane, most of which is monocular (266°). Such wide monocular symmetrical coverage is typically associated with the detection of predators and conspecifics in different directions. Moorhens are exposed to both aerial and terrestrial predators in open water and vegetated margins, and maintenance of near‐panoramic coverage would, therefore, be beneficial. The exceptionally wide cyclopean field may also be advantageous for navigating the structurally complex habitats occupied by moorhens, which typically move through dense emergent vegetation and other visually cluttered environments. Given their relatively small size and skulking behaviour, extensive visual coverage may facilitate the detection of obstacles and conspecifics while moving through a three‐dimensional habitat where visibility is frequently obstructed. The visual field configuration observed here illustrates the well‐established trade‐off between binocular overlap and panoramic surveillance, with moorhens achieving a compromise that supports both predator detection and accurate bill control.

### 4.3 Visual Fields and Head Posture

**FIGURE 3:**
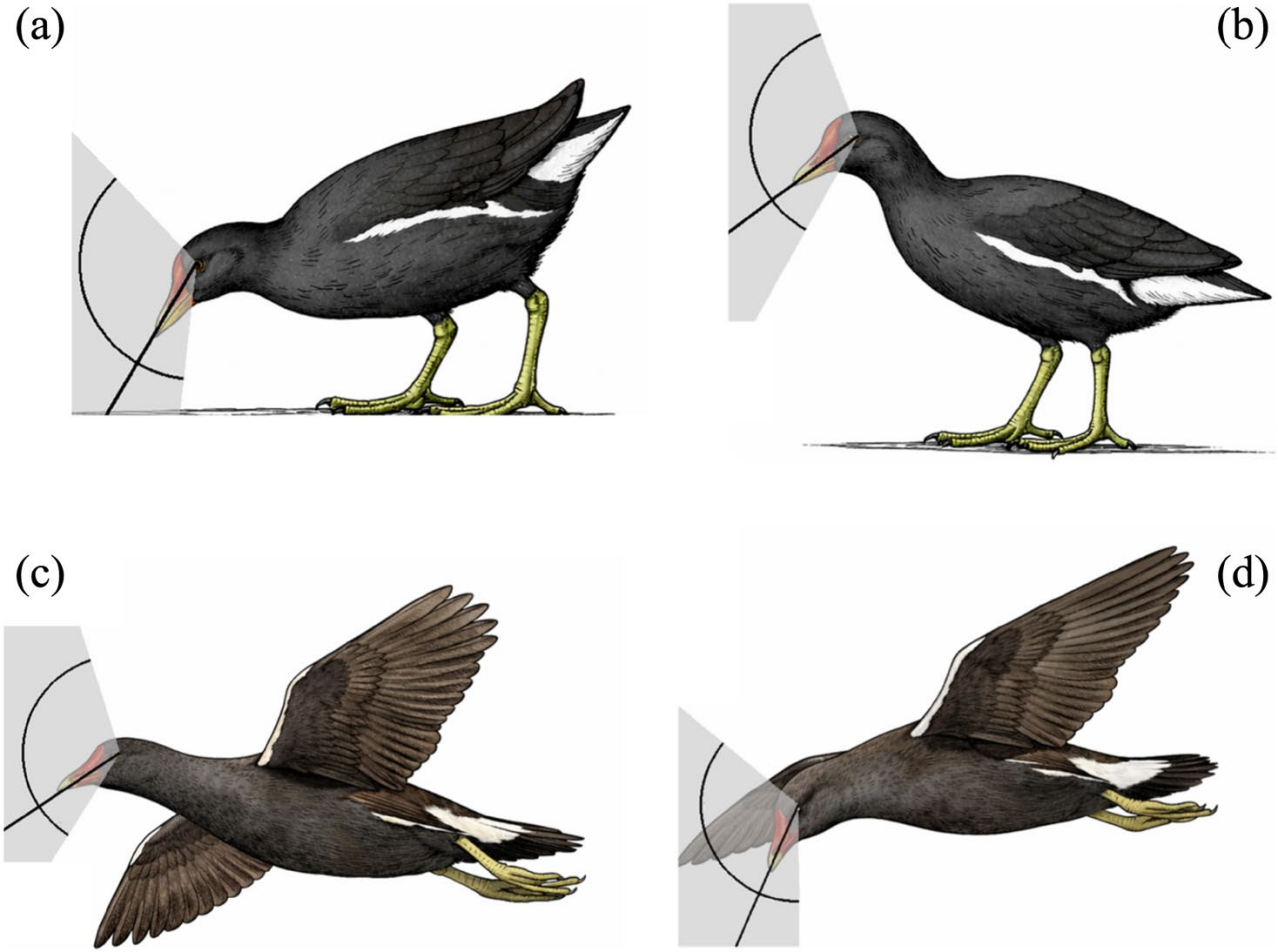
Illustrations of side views of common moorhens in characteristic postures. The projection of a vertical section through the binocular field (as shown in Figure 2f) is superimposed on each illustration and depicted in light grey. (a) In typical foraging pose with the head rotated forwards by 30° the binocular field projects onto the ground below the head. (b) When standing upright the visual field projects in front but not below the head. (c) When in flight and looking ahead the visual field projects to the ground ahead of the bird. (d) When in flight with the head rotated forwards by 30° the bird can maintain a view directly in front of the head and see the ground directly below it. The moorhen was created using a combination of PowerPoint trace function from author photos, photoshop, and sketch‐to‐image in ChatGPT.

The functional consequences of the configuration of the binocular and cyclopean fields are further shaped by posture. Figure 3 presents the visual projection of a vertical cross‐section of the binocular field under various postural conditions on the ground and in flight. When standing upright, the substrate immediately beneath the body lies outside the visual field and becomes visible only when the head is pitched forward by a minimum of 30° compared to the normal resting head posture. This suggests that visual access to the foraging substrate requires active adjustment of head posture. Such movements may represent a dynamic modulation of sensory input, allowing moorhens to alternate between vigilance and substrate inspection, like that seen in blacksmith lapwings ( *Vanellus armatus* ) (Cantlay et al. 2019).

### 4.4 Visual Fields and Structurally Complex Habitats

Birds moving through structurally complex/cluttered environments—characterised by dense vegetation and numerous visual and physical obstacles distributed across multiple spatial planes—must balance obstacle avoidance, predator detection and precise control of pecking movements. The observed visual field design is consistent with the visual demands associated with movement and foraging in structurally complex habitats. In all postures depicted in Figure 3 moorhens retain visual coverage of the space ahead of them. Thus, a moorhen should always be aware of any obstacles in their path when moving through or foraging in structurally complex habitats even if its head is pitched forwards (Figure 3a,d). Such comprehensive coverage of the way ahead does not occur in all birds when the head is pitched forwards. In some species of raptors (Accipitridae), gulls (Laridae), bustards (Otididae), and cranes (Gruidae), pitching the head forwards to examine the ground below results in the birds being effectively blind in their direction of travel (Martin and Shaw 2010; Martin et al. 2012; Cantlay et al. 2024).

### 4.5 Comparison Between the Visual Fields of Blue Cranes and Moorhens

Comparison with the visual fields of blue cranes suggests differences in binocular field configuration that are consistent with broader comparative studies. Because the present study is descriptive and based on a small sample, these comparisons should be interpreted qualitatively rather than as formal statistical tests. Although both blue cranes and common moorhens belong to the order Gruiformes, differences in visual field parameters are evident, chiefly in the extent of binocularity. Maximum binocular field width in blue cranes was 21.5° compared to 34° in the common moorhens. Similarly, vertical extent in blue cranes was considerably smaller than common moorhens, with 75° and 125°, respectively. The blind region directly behind the head was similar in the two species, values of 60.5° and 60°, for blue cranes and common moorhens, respectively. Blue Cranes forage primarily in open habitats and primarily on evasive prey, although their diet does include some sedentary items. Moorhens forage primarily in structurally complex habitats on sedentary items. It has been hypothesised that the taking of evasive prey items, as opposed to static items, primarily drives visual field configurations that result in broader but vertically shorter binocular fields and a broad blind area above the head (Potier et al. 2018), and this seems to be the case in blue cranes. This can result in the birds failing to see ahead when foraging with the head pitched forwards and can render them vulnerable to collisions with human artefacts (Martin and Shaw 2010; Martin 2014; Cantlay et al. 2024). In moorhens pitching the head forward does not result in the loss of visual coverage ahead. This should result in moorhens having low vulnerability to collisions but, more importantly, it will facilitate movement through, and foraging within, structurally complex habitats.

Although based on only two species, the differences between blue cranes and common moorhens are consistent with broader comparative studies (Cantlay et al. 2023; Potier et al. 2018, 2023; Lucas et al. 2024) showing associations between visual field configuration and foraging ecology. The present results therefore extend the taxonomic coverage of visual‐field data within Gruiformes and provide additional descriptive evidence compatible with the hypothesis that ecological factors contribute to variation in avian visual field configuration (Martin 2017a).

## Author Contributions

**Steven J. Portugal:** conceptualization (equal), data curation (lead), formal analysis (lead), funding acquisition (lead), investigation (equal), methodology (equal), project administration (lead), resources (lead), supervision (lead), validation (lead), visualization (equal), writing – original draft (lead), writing – review and editing (lead). **Eleanor A. Lucas:** conceptualization (equal), data curation (supporting), investigation (lead), methodology (equal), project administration (supporting), writing – review and editing (supporting). **Simon Potier:** formal analysis (supporting), methodology (supporting), validation (supporting), visualization (supporting), writing – review and editing (supporting). **Gérard Rocamora:** project administration (supporting), resources (equal), validation (supporting), writing – review and editing (supporting). **Graham R. Martin:** conceptualization (supporting), data curation (supporting), formal analysis (supporting), validation (supporting), visualization (equal), writing – review and editing (supporting).

## Funding

The authors have nothing to report.

## Conflicts of Interest

The authors declare no conflicts of interest.

## Data Availability

All data are available within the paper.
